# Cerebrolysin adjuvant treatment in Broca's aphasics following first acute ischemic stroke of the left middle cerebral artery

**Published:** 2010-08-25

**Authors:** DC Jianu, DF Muresanu, O Bajenaru, BO Popescu, SM Deme, H Moessler, SZ Meinzingen, L Petrica, M Serpe, S Ursoniu

**Affiliations:** * Department of Neurology, ‘Victor Babes’ University of Medicine and Pharmacy, Clinical Emergency County Hospital, Timisoara Romania; ** Department of Neurology, ‘Iuliu Hatieganu’ University of Medicine and Pharmacy Cluj NapocaRomania; *** Department of Neurology, ‘Carol Davila’ University of Medicine and Pharmacy, University Hospital, BucharestRomania; **** Department of Neurology, ‘Vasile Goldis’ West University AradRomania; ***** Ever Neuro Pharma, UnterachAustria; ****** Department of Internal Medicine, ‘Victor Babes’ University of Medicine and Pharmacy, Clinical Emergency County Hospital, Timisoara Romania; ******* Department of Internal Medicine, ‘Victor Babes’ University of Medicine and Pharmacy, Clinical Emergency County Hospital, Timisoara Romania

## Abstract

Background: The aim of our study was to assess the efficacy of Cerebrolysin administration in Broca's aphasics with acute ischemic stroke.

Methods: We registered 2,212 consecutive Broca's aphasics following an acute ischemic stroke admitted in four departments of neurology in Romania, between September 2005 and September 2009. Language was evaluated with the Romanian version of the Western Aphasia Battery (WAB). The following inclusion criteria were used for this study: age 20%75 years, admission in the hospital within 12 hours from the onset of the symptoms, diagnosis of first acute left middle cerebral artery (MCA) ischemic stroke, presence of large artery disease (LAD) stroke, a NIHSS score of 5%22 points, and a therapeutic time window within 72 h. Fifty two patients were treated with Cerebrolysin (Cerebrolysin group) as an adjunctive treatment. A placebo group, which received saline infusions (n=104 patients) were matched to the NIHSS and WAB scores, gender and age of the Cerebrolysin group at baseline. We assessed spontaneous speech (SS), comprehension (C), repetition (R), naming (N), and Aphasia Quotient (AQ) scores of the two groups in an open label design, over 90 days, the mRS scores and mortality.

Results:  The Cerebrolysin and the placebo groups had similar age (66+/%8 versus 65+/%8 years) and sex ratio (14/38 versus 30/74). The mean AQ scores and the mean subscores for 3 subtests of WAB (SS, R, N) were similar at baseline and improved in the Cerebrolysin group significantly (p<0.05) over placebo group at all study time points. The mRS score at 90 days was also lower in the Cerebrolysin group than in the placebo group. Cerebrolysin and placebo were both tolerated and safe, and no difference in the mortality rate was seen (3.8% in each group).

Conclusion: Cerebrolysin is effective for the treatment of Broca's aphasics with a first acute ischemic stroke of the left MCA territory.

## Background and purpose

The aim of the treatment in acute ischemic stroke is to limit the damage and to improve recovery by reperfusion and neuroprotection. Thrombolysis is restricted to a limited proportion of selected patients, and this has led to the investigation of alternative approaches, especially neuroprotection (Broderick 2000, Ferro 2006, Fisher 2006, Hacke 2006). Neuroprotective drugs aim to salvage the penumbra, limit the infarction size, prolong the time window for reperfusion therapy, and/or minimize postischemic reperfusion injury, the inflammation and the risk of haemorrhage (Gutierrez 2000, Rodriguez%Yanez 2006, Serena 2006). Neuroprotective therapies have been effective in experimental models of ischemia, but, now, there is no definitive evidence of benefit in the numerous trials carried out on humans, although some subgroups of patients seem to benefit from some of them (De Keyser 1999, Martinez%Villa 2001, Ovbiagele 2003, Labiche 2004, Lizasoain 2006). Cerebrolysin (Cere) is a compound with neurotrophic and neuroprotective activities, which has shown promise in earlier experimental and clinical stroke studies. It is produced by enzymatic breakdown of purified brain proteins and consists of biologically active low molecular weight peptides and free aminoacids (Schwab 1997, Parsons 2000, Saton 2000, Frey  2002, Schauer 2006). Several European small trials have suggested that Cerebrolysin improves motor function, cognitive performance and global function of acute stroke patients (Gusev 1994, Barolin 1996, Haffner 2001, Ladurner 2001, 2005, Muresanu 2004, Skvortsova 2004). The current study was conducted to assess the efficacy of an adjuvant administration of Cerebrolysin in Broca's aphasics following a first acute ischemic stroke of the left middle cerebral artery (MCA).

## Patients and methods

We conducted a randomized clinical trial from September 2005 to September 2009, in which departments of neurology from 4 cities of Romania participated. A total of 2,212 consecutive right handed patients with Broca's aphasia (native Romanian speakers), with an acute left middle cerebral artery territorial infarction, were entered in this study. Before the stroke, all the 2,212 right%handers showed the preference to use the right hand in all one%handed tasks and, in bimanual tasks, executed the more precise movements with the right hand (data obtained from their relatives) (Lecours 1988, Viader 2002).

All the registered patients underwent a careful medical history, physical and neurological examination, routine blood examinations, urinanalysis, electrocardiogram, chest X%rays, and nonenhanced brain CT scans, at admission. Particular attention was given at admission to language function, which was evaluated by means of a Romanian version of the Western Aphasia Battery (WAB) (Kertesz 1980, Kory Calomfirescu 1996). 

In addition, the following data were assessed in all of the patients, by using common data%sheets: (1) age and gender; (2) time from onset to hospital arrival; (3) a history of stroke (including Broca's aphasia); (4) site and number of acute lesions on CT and MRI; (5) stroke subtype (clinical category); (6) National Institutes of Health Stroke Scale (NIHSS) score on admission.

The clinical categories were defined by using clinical and radiographic diagnosis rubrics according to the Classification of Cerebrovascular Diseases Ⅲ, developed by the National Institute of Neurological Disorders and Stroke (1990). The main subtypes included: (1) large artery disease (LAD) was presumed in patients with significant stenosis (>50%) of the lumen diameter or occlusion of the main trunk of the left internal carotid artery (ICA); (2) cardio embolism (CE) mainly included atrial fibrillation, left ventricular dyskinetic segment, recent myocardial infarction with dyskinetic segment, intracardiac thrombus or tumour, cardiomyopathy, and other less common sources, (3) small artery disease was presumed in patients with lacunar infarcts and in patients with hypertension, and/or diabetes mellitus in the absence of LAD and CE; (4) mixed etiology was presumed in the coexistence of LAD, CE and/or small artery diseases; (5) other strokes (Hacke 2006, Masdeu 2006).

Conventional Magnetic Resonance Imaging (MRI), Duplex Sonography of the carotid and vertebral arteries (My LAB 50 ESaote), transcranial Doppler (Explorer CVS%DMS systems), Magnetic Resonance angiography (MRA), and transthoracic echocardiography (TTE) were performed. MRI of the brain was performed on General Electric Medical System % Signa Horizon Lx 1.0 T, software Signa Lx versio 9.0. The standardised MRI protocol consisted of axial T_2_%weighted images with fluid attenuated inversion recovery (FLAIR), coronal T2%weighted images and sagittal T1%weighted images. The vascular distributions of the infarcts were determined with templates of cerebral vascular territories. The measurement of stenosis on MRA was computed directly on the maximum intensity projection and collapsed views. Results that were >50% were considered to constitute significant stenosis.

From these 2,212 Broca's aphasics registered, we selected eligible patients, who met the following criteria:

- 20%75 years of age.
- Admission within 12 hours from the onset of the symptoms.
- A diagnosis of first acute ischemic stroke in the territory of the left MCA (CT and MRI were performed within the first 48 hours from the symptoms' onset). We excluded the patients with baseline brain CT scan with hypodensity or mass effect involving greater than 50% of the left MCA territory. However, loss of gray%white differentiation, sulcal effacement, and other early CT changes that have high inter%rater variability were not exclusion criteria (Masdeu 2006). Selected patients had to have on MRI examination at admission a single large (>20 mm diameter) ischemic lesion in the territory of the left MCA.
- We excluded the patients with large artery disease (LAD) and additional clinical small vessel abnormalities revealed by FLAIR or T_2_%weighted MRI slices. A control MRI was performed 3 months later for all the selected patients who survived, in order to identify an eventual new cerebral lesion. All of them remained with a single lesion at 90 days.
- The presence of a large artery disease (LAD) stroke (Doppler ultrasonography, and transthoracic echocardiography were performed within 48 hours from the symptoms' onset).
- A National Institute of Health Score Scale (NIHSS) score of 5%22 points on admission. A senior neurologist from each department determined the NIHSS score, independently of Doppler ultrasonography and neuroimaging results.

Exclusion criteria were the following: acute myocardial infarction, preceding dementia, other severe concomitant diseases (renal and/or hepatic insufficiency), participation in other studies. Concomitant use of nootropic drugs (e.g. piracetam), drugs with dilating effects on cerebral blood vessels, as well as chronic intake of anti%depressants, tranquilizers, sedatives or CNS stimulants were prohibited (Haffner, 2001). 

Eligible patients were divided into three groups:

- Cerebrolysin group. The patients received standard therapy and adjuvant therapy with Cerebrolysin within a therapeutic time window shorter than 72 hours;
- Placebo group. Patients received standard therapy and placebo treatment within 72 hours from stroke onset. They were matched to the Cerebrolysin group patients, with respect to age, gender, NIHSS and WAB scores.
- The third group. Patients received only standard therapy within 72 hours from stroke onset. Their evolution will be reported separately.

The local ethics committees approved the protocol of the study, and a written informed consent was obtained for all the selected patients from their relatives. Immediately after the selection of the two groups and the baseline evaluation, patients were put on study medication, once daily i.v. infusions (in a peripheral vein over a period of 20 minutes) of active medication or placebo for 21 consecutive days. The active medication contained 30 ml of Cerebrolysin mixed with 70 ml of normal saline. Placebo contained 100 ml of normal saline. In addition to the study treatment, both the active and the control group patients received standard therapy, including acetylsalicylic acid (ASA) (250 mg/day, p.o.) which was continued during the follow%up examination on day 90 and beyond.

### Efficacy assessment

Seven evaluation visits were scheduled for the selected patients of the two groups: at baseline (day 0) and at all subsequent study visits, on study days 1, 3, 7, 14, 21 (the last day of active treatment), and day 90. Patients were evaluated by physicians who were unblinded to treatment group allocation.

### Study objectives

The aim of the study was to investigate the following issues in Broca's aphasics following the first acute ischemic stroke of the left MCA:

- Primary end point: the effects of Cerebrolysin in comparison with placebo in language performance in all subsequent study visits, on days 1, 3, 7, 14, 21 and 90.
- Secondary end points:
  1. % to assess clinical outcome at 90 days between Cerebrolysin group and placebo group;
  2. % to compare the mortality rate between the two groups at 90 days;
  3. % to determine the safety of Cerebrolysin treatment: emergent adverse events (AEs).

The following clinical evaluation scales were used:

1. the National Institutes of Health Stroke Scale (NIHSS) (at baseline);
2. the Romanian version of the Western Aphasia Battery (WAB), (Kertesz 1980, Kory Calomfirescu 1996), which evaluated the language function. WAB included the assessment of spontaneous speech (SS), comprehension (C), repetition (R) and naming (N). We have calculated the scores and have established the aphasia quotient (AQ) for all the 7 visits (Table 1);
3. the modified Rankin Scale (mRS) score was used to assess the clinical outcome at 90 days between the 2 groups;
4. we compared the mortality rate between the two groups at 90 days.

**Table 1:** The Aphasia Quotient (AQ)

Subscale	Maximum score
Spontaneous Speech (SS)	
Functional content (SSFC)	10
Fluency (SSFL)	10
SS score=SSFC+SSFL	10+10=[20]*
Comprehension (C)	
Yes/no questions (CYN)	60
Auditory word recognition (CWD)	60
Sequential commands (SCO)	80
C score=(CYN+CWD+SCO)/20	(60+60+80)/20=[10]*
Repetition (R) score	100/10=[10]
Naming	
Object naming (OBNA)	60
Word fluency (WDFL)	20
Sentence completion (SECO)	10
Responsive speech (RESP)	10
N score=(OBNA+WDFL+SECO+RESP)/10	(60+20+10+10)/10=[10]*
APHASIA QUOTIENT= AQscore=(SSscore+Cscore+Rscore+Nscore)x2	Add above totals in [...] in rows marked by *, then x2 =(20+10+10+10)x2=100

The efficacy of Cerebrolysin was evaluated based on the functional and the language function assessment of the patients. (Lecours 1988, Viader 2002).

The evolution of motor functions, cognitive performances, and global clinical impressions will be reported separately.

### Statistical analysis

The comparison of the two groups (Cerebrolysin group and placebo group) with regard to demographic and background characteristics was assessed by using descriptive statistics and appropriate parametric and non%parametric statistical tests. Data from Visit 1 (day 0) were used for the primary analysis of spontaneous speech (SS), comprehension (C), repetition (R), and naming (N) subscores, and Aphasia Quotient (AQ) total scores. The secondary analysis included the data on days 1, 3, 7, 14, 21 (the last day of the active treatment), and day 90. The mean score at the specific visit and the mean score change from baseline visit for all efficacy measures, were analysed. Student's t%test or non%parametric Mann%Whitney U test were used in the comparison of continuous variables between the groups, total scores and score changes from baseline as appropriate. Because the tests for equality of variance proved a significant difference, in the selected cases (e.g. A Q at visit 2%day 0, or SS at visit 6%day 21) we used modified t%test with Welch's correction. Efficacy variables were analysed by using two%tailed statistics. We compared the mRS scores and mortality between the two groups. We used the chi test for favourable outcome (mRS score 0%2) and mortality. A p value <0.05 was considered statistically significant in all tests.

STATA VERSION 9.2 software was used for all calculations.

## Results

**Figure 1:**
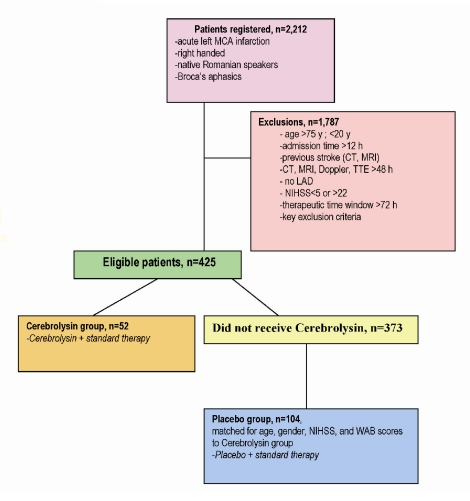
Flow chart showing process of patient selection; Legend: MCA–middle cerebral artery, CT–computer tomography, MRI–magnetic resonance imaging, Doppler–Duplex carotid and vertebral Sonography, TTE–transthoracic echocardiography, LAD–large artery disease, NIHSS–National Institute of Stroke Scale, WAB–Western Aphasia Battery.

From 2,212 patients registered, 425 were eligible (Figure 1).

From the 425 eligible patients, fifty%two patients met the criteria of inclusion in the Cerebrolysin group, and one hundred and four patients were selected for the Placebo group (Figure 1).

### Baseline characteristics of the two groups

Table 2 shows the baseline characteristics of the Cerebrolysin group (n=52) and placebo group (n=104).

**Table 2:** Characteristics of the two groups (Cerebrolysin group, and placebo group) at baseline; Legend: F–female, M–male, SD–standard deviation, SS–spontaneous speech, C–comprehension, R–repetition, N–naming, AQ–aphasia quotient, mRS–modified Rankin Scale.

Patients	Cerebrolysin group (n=52)	Placebo/group (n=104)	p value
Gender, F/M	14/38	30/74	0.801
Age, years (mean+/% SD)	66+/%8	65+/%8	0.462
The median NIHSS score on admission	14	14	1.000
Interval time from stroke onset to hospitalization, h (mean +/% SD)	8.2+/%2.4	7.8+/%2.6	0.354
Interval time from stroke onset to active or placebo treatment, h, (mean+/% SD)	38.3+/%10.2	39.4+/%11.1	0.550
Initial SS scores (mean+/% SD)	4.09+/%1.89	4.13+/% 1.91	0.901
Initial C scores (mean+/% SD)	8.52+/%0.51	8.49+/%0.50	0.726
Initial R scores (mean+/% SD)	3.91+/%0.87	3.87+/%0.86	0.785
Initial N scores (mean+/% SD)	4.12+/%0.98	4.07+/%0.97	0.762
Initial AQ scores (mean+/% SD)	41.28+/%6.67	41.12+/%6.59	0.887
mRS <2 ,(%) at baseline	19.2% (n=10)	19.2% (n=20)	1.000

The most common sites of single infarcts in the territory of the left MCA of the 156 Broca's aphasics selected were left inferior frontal, left inferior parietal and /or left insula (44 patients%84.6% of the Cerebrolysin group and 90 patients %86.5% of the placebo group). In other cases, there was a deviation from the classic clinical%anatomic correlations of Broca's aphasia: left superior temporal: 8 patients in the Cerebrolysin group and 14 patients in the placebo group. (Lecours 1988, Basso 1995, Kreisler 2000).

**Table 3:** Aphasia, cerebral infarcts and stroke etiology

Group of pts	Cerebral infarcts	Number of pts n (%)	P%value	Large artery disease	Number of pts n (%)	P%value
Cerebrolysin group n=52	Left inferior frontal; left inferior parietal; left insula	44 (84.6%)	0.745	Ipsilateral or bilateral ICA diseases (extra, and/or intracranial stenosis or occlusions)	48 (92.3%)	0.816
			ICA diseases+MCA diseases	3 (5.8%)	
	Left superior temporal	8(15.4%)	MCA diseases	1 (1.9%)	
placebo group n=104	Left inferior frontal; left inferior parietal	90(86.5%)	Ipsilateral or bilateral ICA diseases (extra, and/or intracranial stenosis or occlusions); ICA diseases+MCA diseases	9 (8.7%)	
	Left superior temporal	14 (13.5%)		MCA diseases	2 (1.9%)	

Primary end%point: the effects of Cerebrolysin in comparison with placebo in language performance in all subsequent study visits, on days 1, 3, 7, 14, 21, and 90.

### The evolution of Broca's aphasia

**Figure 2:**
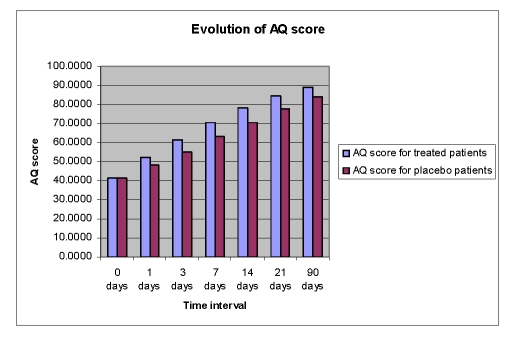
Time course of AQ score from day 0 to day 90 in the Cerebrolysin group and in the placebo group; Legend: AQ score=aphasia quotient score= SS score+C score+R score+N score)x2

**Figure 3:**
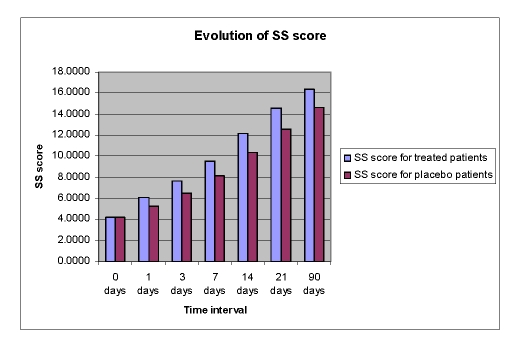
Time course of SS score from day 0 to day 90 in the Cerebrolysin group and in the placebo group; Legend: SS score=spontaneous speech score= SSFC (functional content) score+SSFL  (fluency) score

**Figure 4:**
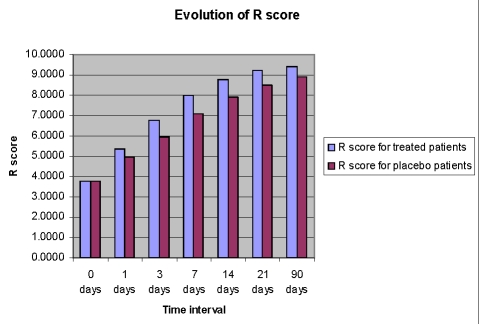
Time course of R score from day 0 to day 90 in the Cerebrolysin group and in the placebo group Legend: R score=repetition score/10 +WDFL (word fluency) score+SECO (sentence completion) score+RESP (responsive speech) score]/10

**Figure 5:**
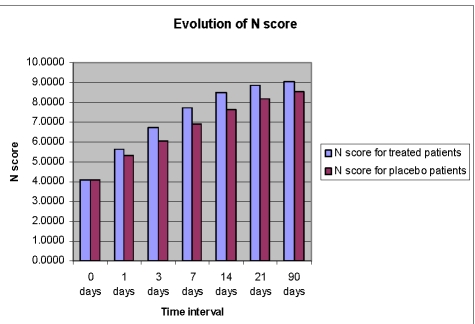
Time course of N score from day 0 to day 90 in the Cerebrolysin group and in the placebo group; Legend: N score=naming score=[OBNA (object naming) score

The mean AQ scores and the mean scores for 3 subtests of WAB (Spontaneous Speech%SS, Repetition%R, and Naming%N) improved over time in both groups, but the Cerebrolysin group had a significant improvement (p<0.05) over the placebo group (Figure 2,Figure 3,Figure 4,Figure 5).

**Figure 6:**
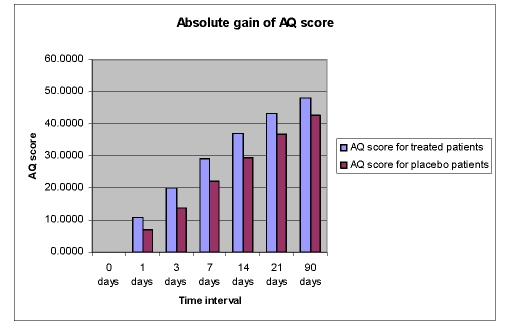
Absolute gain of AQ score

**Figure 7:**
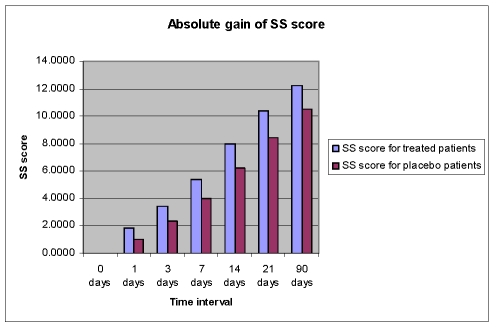
Absolute gain of SS score

**Figure 8:**
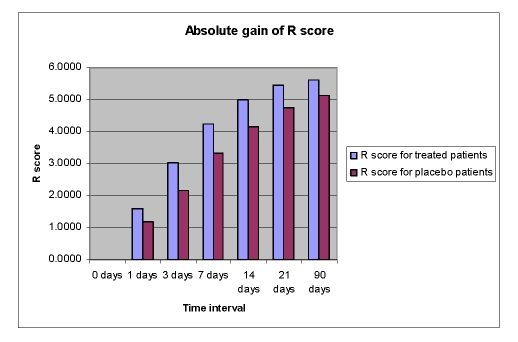
Absolute gain of R score

**Figure 9:**
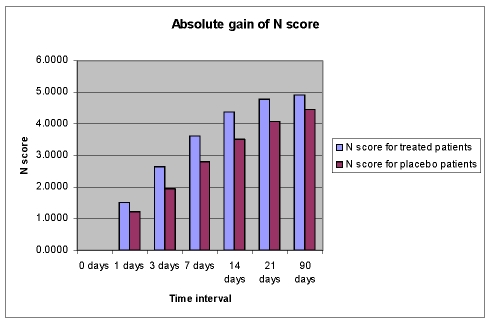
Absolute gain of N score

This improvement of language functions was evident at all study time points, but the drug%placebo difference in favour of Cerebrolysin was most pronounced during the first 7 days of treatment (Figure 6,Figure 7,Figure 8,Figure 9).

Interestingly, the effect of Cerebrolysin was maintained after cessation of the treatment up to the 21%day assessment, during the overall period of 90 days. 

**Figure 10:**
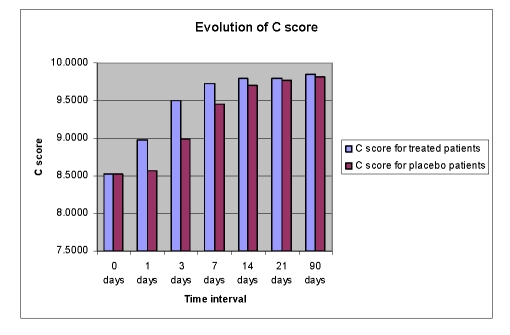
Evolution of C scores

**Figure 11:**
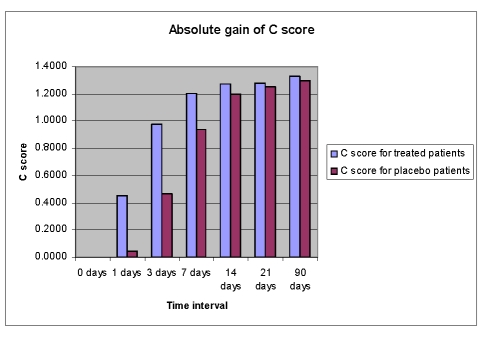
Absolute gain of C score; Legend: C score=comprehension score=[CYN (yes/no questions) scores+CWD (auditory word recognition) scores+SCO  (sequential commands)score]/20

No significant treatment differences have been noticed with the Comprehension subtest of WAB. The baseline scores for both groups (8.52 for Cerebrolysin and 8.49 for placebo) were very close to the maximum score for Comprehension (10.0) (Figure 10, Figure 11).

**Figure 12:**
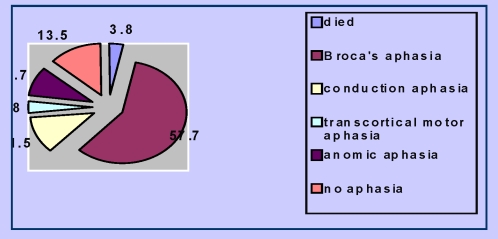
The evolution of language functions in the Cerebrolysin group (n=52 patients) on day 90

**Figure 13:**
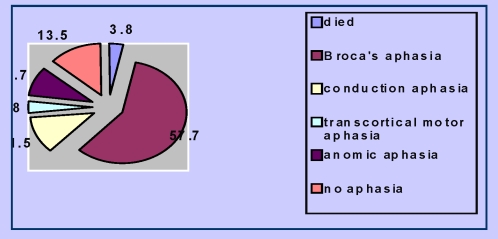
The evolution of language functions in the placebo group (n=104 patients) on day 90

At the final evaluation (day 90), the evolution of language functions in the Cerebrolysin group was more favourable (Figure 12, Figure 13):

Secondary end%points:

1. Clinical outcome at 90 days in Cerebrolysin group and placebo group. Patients with favourable outcome (mRS <2 at 90 days) were more frequently observed in the Cerebrolysin group than in the placebo group (16 patients%38.09% were added to the initial 10 patients with mRS<2 in the Cerebrolysin group, versus 24 patients%28.57% who were added to the initial 20 patients with mRS<2 in the placebo group P=0.279).
2. Mortality at 90 days in Cerebrolysin group and placebo group. However, no difference between the two groups was seen in the mortality rate (3.8% in each group); during the overall period of 90 days. Two patients died in the Cerebrolysin group, (one patient with pulmonary embolism, one patient with pneumonia) and four patients in the control group (two patients with pulmonary embolism, two patients with pneumonia). The investigators considered no relation with the investigational drug in all 6 cases of death.
3. Safety: Cerebrolysin and Placebo were both well tolerated and safe. The overall incidence of AEs was similar in both groups (8 patients%15.4% of Cerebrolysin group as compared to 11 patients%10.6% of placebo group experienced at least one AE; P=0.387)

The most common AEs reported in the Cerebrolysin group was hypertension; it occurred with a similar frequency in the placebo group (2 patients%3.8% versus 4 patients%3.8%, respectively P=1.000). Overall, there was no significant difference between the two groups with regard to the frequency or nature of adverse events. Most AEs were mild to moderate in severity, and were classified as not related to the investigational drug.

## Discussion

Several small trials suggested that Cerebrolysin, administered as a continuous infusion for 21 days improves motor function and global function, compared to placebo (Gusev 1994, Barolin 1996, Haffner 2001, Ladurner 2005). Larger clinical trials would be required to establish the value of continued neuroprotection and to determine the pharmacokinetics/dynamics of Cerebrolysin (Labiche, Grotta, 2004, Muresanu 2007). 

Future neuroprotection clinical trials need a new design concerning stroke type, stroke severity, identifying the ischemic penumbra, time window for drug administration, combination therapy, dosing regimen, statistical power, and study outcomes (Ovbiagele 2003, Muresanu 2007). Thus, patients with lacunar strokes frequently recover spontaneously, therefore, only patients with LAD were included in our study.

The assessment of cognitive improvement in stroke trials has received little attention in the past, where studies have clearly concentrated on the neurological and functional outcome. This has, perhaps, been partly due to an underestimation of the importance of the cognitive function, especially the language function, as a predictor of rehabilitation outcome after stroke (Muresanu 2004, Ladurner 2005). In the present study, we tried to further explore the clinical effectiveness of Cerebrolysin in acute ischemic stroke with regard to language function recovery.

Outcome measures lump together different disabilities (e.g: sensorimotor, cognitive) that may respond differently to treatment % thus obscuring treatment effects. Because of this, we assessed only language functions in a single type of aphasia: Broca's aphasia (Jianu 2001).

Broca's aphasia represents a primary deficit in language output, with a relative preservation of comprehension. The speech production of our patients was slow, as compared to normal; the prosody was greatly reduced, with significant articulatory disturbances and word finding difficulties. Repetition and writing were impaired.

Broca's aphasia is most often present due to a vascular lesion, with frontal suprasylvian localisation, especially an embolic infarction (LAD), less often atherosclerotic thrombus in the territory of the superior division of the middle cerebral artery (MCA) (Viader 2002). For this reason, we selected only patients with left MCA infarctions restricted to the upper division of the left MCA, and not complete left MCA stem occlusion or occlusion of the left ICA (which produces global aphasia).

Because of the distribution of the superior branch of the MCA, Broca's aphasia was frequently associated with a right side facio%brachial paresis and disorders of sensation. In consequence, the baseline NIHSS scores were relatively homogeneous for Broca's aphasics in the Cerebrolysin group and the placebo group. 

Initial Aphasia Quotient scores were relatively homogeneous at baseline in the two groups because all Broca's aphasics presented comprehension scores between 8 and 10, and repetition, naming, and spontaneous speech scores between 2 and 5.

Consequently, at baseline, there were no significant statistical differences between the two groups. For all these reasons, we did not use any adjustments at the beginning of the study, and the homogeneity for our eligible patients was significant in order to match the placebo group with the Cerebrolysin group.

Our study clearly demonstrates that i.v. adjuvant treatment with Cerebrolysin results in statistically significant and clinically important improvements of language function in patients with Broca's aphasia with a first acute ischemic stroke. We have demonstrated a clear improvement of Aphasia Quotient score and of Spontaneous Speech (SS), Repetition (R), and Naming (N) subscores in our patients sample after Cerebrolysin treatment during the first 7 days after the stroke onset, indicating a fast onset of action of Cerebrolysin, offering the possibility for an earlier rehabilitation (Ladurner 2005).

Our patients were characterised by mild baseline impairment of the comprehension subtest of WAB. The average score was close to the maximum test score for this scale. Evidently, such a situation induces a pronounced ceiling effect, which thereby impedes a measurable treatment effect for the test substance.

In comparison with the placebo treatment, a significant effect of Cerebrolysin on language function recovery was still evident on day 90, after cessation of active treatment on day 21. These data indicate a possible disease modifying action and stabilising effect of Cerebrolysin, which goes beyond a purely symptomatic effect. This long%term stabilising effect can be attributed to a potent neuroplasticity inducing effect of Cerebrolysin.

Patients from the Cerebrolysin group had an increased significant frequency of good outcomes, than the patients of the placebo%group. 

Cerebrolysin and placebo were both well tolerated and safe, and no difference in the mortality rate was observed between patients with and without Cerebrolysin therapy.

### Our study had some limitations

The therapeutic time window for inclusion in our study was of 72 hours after the onset of the ischemic stroke. It is generally accepted that early commencement of neuroprotective therapy (within the first 6 hours) favours more complete recovery of the impaired neurological functions (Ferro 2006, Gutierrez 2006, Schauer 2006). However, none of our patients has been treated within this therapeutic time window. 

MRI was used in our study conventionally; in comparison with this technique,  diffusion%weighted imaging (DWI) is more sensitive to the presence of small new ischemic lesions or reversible ischemic lesions, hours after the onset of the stroke, and it is able to differentiate recent ischemic lesions from old ones or nonspecific white matter high%signal intensities (Masdeu 2006). DWI was also reported to be superior to conventional MRI in the detection of multiple ischemic lesions (Hacke 2006, Masdeu 2006).

Moreover, physicians who assessed patients' outcome were not blinded to treatment. Therefore, it is possible that the efficacy of Cerebrolysin therapy is overestimated. 

In conclusion, despite the limitations associated with the small number of patients in our study compared to other stroke studies, the mild baseline impairment of comprehension, the wide time window of active treatment and the use of conventional MRI instead of DWI, our study suggests a therapeutic effect of Cerebrolysin in acute ischemic patients, in particular with regard to language function recovery. 
